# Secretory Products in Petals of *Centaurea cyanus* L. Flowers: A Histochemistry, Ultrastructure, and Phytochemical Study of Volatile Compounds

**DOI:** 10.3390/molecules27041371

**Published:** 2022-02-17

**Authors:** Aneta Sulborska-Różycka, Elżbieta Weryszko-Chmielewska, Beata Polak, Beata Stefańczyk, Anna Matysik-Woźniak, Robert Rejdak

**Affiliations:** 1 Department of Botany and Plant Physiology, University of Life Sciences, Akademicka 15, 20-950 Lublin, Poland; elaweryszko@wp.pl; 2 Department of Physical Chemistry, Medical University of Lublin, Chodźki 4 A, 20-093 Lublin, Poland; beata.polak@umlub.edu.pl (B.P.); office.stefanczyk@gmail.com (B.S.); 3 Department of General Ophthalmology, Medical University of Lublin, Chmielna 1, 20-079 Lublin, Poland; annawozniak@umlub.pl (A.M.-W.); robert.rejdak@umlub.pl (R.R.)

**Keywords:** cornflower, ray florets, volatile compounds, sesquiterpenes, localization of secretion, cuticular pattern

## Abstract

(1) Background: *Centaurea cyanus* L. is a medicinal plant whose flowers are widely used in herbal medicine. The aim of the study was to localise flower tissues that are responsible for the production of secretory products in petals and to analyse the volatile compounds. The volatile compounds of the flowers of this species have not been investigated to date. (2) Methods: Light, fluorescence, scanning and transmission electron microscopy techniques were used in the study. Lipophilic compounds were localised in the tissues using histochemical assays. Volatile compounds were determined with the use of solid phase microextraction (SPME) and gas chromatography-mass spectrometry (GC-MS). (3) Results: The study showed production of secretion in the petal parenchyma, whose ultrastructure has features of a secretory tissue. The lipophilic secretion was localised in the cells and intercellular spaces of the parenchyma and in the walls and surface of epidermal cells, where it accumulated after release through cuticle microchannels. Sesquiterpenes were found to constitute the main group of volatile compounds, with the highest content of β-caryophyllene (26.17%) and α-humulene (9.77%). (4) Conclusions: Given the presence of some volatile components that are often found in resins (caryophyllene, delta-cadinene) and the abundant secretion residues on the epidermal surface, we suppose that the *C. cyanus* secretion released by the flowers is a resinaceous mixture (oleoresin), which is frequently found in plants, as shown by literature data. This secretion may play an important role in the therapeutic effects of *C. cyanus* flowers.

## 1. Introduction

Plants emit different volatile organic compounds from their aerial and underground parts [1]. Most often they are produced by leaves and flowers of aromatic plants [2]. In flowers, volatile compounds are primarily synthesized in epidermal cells of petals, from which they are directly released into the atmosphere [1]. Volatile organic compounds serve various functions in flowers: attraction of pollinators or defence against herbivores and pathogens [3]. Secretory products of flowers are natural multi-component mixtures of volatile and non-volatile products. Terpenes, terpenoids, and molecules with an aromatic ring are present at high concentrations [4,5,6]. Volatile and semi-volatile compounds that are part of essential oils can constitute 85–99% of the entire oil fraction. Among them, there are hydrocarbon and derived mono-and sesquiterpenes, aliphatic and olefinic C_6_–C_12_ non-terpene aldehydes, alcohols, ketones, esters, and acids, along with several aromatic compounds. Among these compounds, mainly terpenes are responsible for the medicinal, culinary, and fragrant uses of aromatic plants [7]. Volatile compounds comprise primarily monoterpenes (C_10_) and sesquiterpenes (C_15_) [3,7,8]. The non-volatile residue is mainly composed of flavonoids, coumarins, diterpenoids, sterols, and fatty acids [8].

Monoterpenes and sesquiterpenes play an important role in the ecological relationships between plants and other organisms. These compounds are involved in the formation and emission of floral scents during flowering and in plant response to damage during herbivore attacks [9]. Monoterpenes may be responsible for the allelopathic properties of plants and facilitate their adaptation to abiotic stresses [2]. Sesquiterpenes belongs to semi-volatile organic compounds [1]. They have various properties, as they exert antitumor, antiviral, cytotoxic, immunosuppressive, phytotoxic, antifungal, insect antifeedant, and hormonal effects [10].

Fragrant volatile organic compounds are synthesised, accumulated, and emitted into the atmosphere by specialised secretory cells, i.e., osmophores, papillate cells, glandular trichomes, ducts, and cavities [9,11,12]. Asteraceae plants are rich in this kind of products [9] and are characterised by the presence of external and internal secretory tissues, including various types of glandular trichomes and secretory ducts differing in their structure [13]. Many represetatives of Asteraceae family have secretory ducts in roots, stems, leaves, and bracts, but not in petals [14,15,16]. The presence of such structures in petals has been detected in only few taxa, e.g., in *Ambrosia* [17] and *Tagetes* [18]. Chiru et al. [19] observed secretory ducts in *C. cyanus* stems and leaves and classified them as latex vessels. The authors detected protective trichomes on the surface of leaves and bracts. Haratym et al. [20] reported the presence of secretory ducts in stems and leaves, glandular trichomes on stems and bracts, and non-glandular trichomes on stems, leaves, and bracts in this species.

To date, the chemical composition of essential oils, but not volatile compounds, in the aerial parts of several tens of *Centaurea* taxa representing medicinal plants has been studied. Many of these plants originate from Turkey [21,22,23,24,25] and several species were analysed in Croatia [26,27], Iran [28], Spain [27], and Egypt [29]. The large variation in the quantity and quality of compounds contained in these oils has been shown to depend on the species, genetic traits, and geographic factors [23,24,29,30]. The research conducted by the abovementioned authors indicates that sesquiterpenes are the dominant group of compounds contained in essential oil from aerial parts of various *Centaurea* species. Analyses of essential oil extracted from aboveground parts of *C. cyanus* in Turkey revealed that carvacrol (25.5%) and hexadecanoid acid (6.4%) were its main constituents [31]. The composition of the volatile compounds from *C. cyanus* flowers, i.e., a valuable medicinal raw material, has not been investigated to date.

The aim of the present study was to identify tissue structures in *C. cyanus* ray florets involved in the production and release of secretory products containing volatile compounds. A special focus was placed on the localisation of secretion in tissues using fluorescence microscopy and histochemical assays. We also studied the ultrastructure of the epidermis and parenchyma in flower petals to demonstrate potential secretory properties of these tissues. Given the biological properties of volatile compounds and their important role in the interaction with the environment (e.g., attraction of pollinators or defence against agrophages), we have conducted phytochemical analyses facilitating determination of these compounds in *C. cyanus* flowers and thus filled the gap in this knowledge.

## 2. Results

### 2.1. Micromorphology of Ray Florets

**Figure 1:**
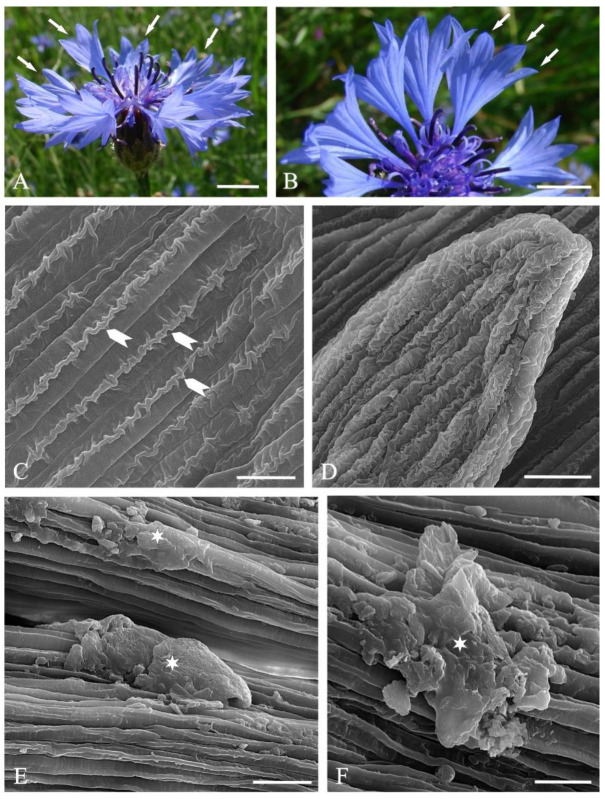
Inflorescences of (**A**,**B**) *Centaurea cyanus* L. and micromorphology of the epidermis surface in ray florets (**C**–**F** SEM): (**A**) head inflorescence composed of disc florets in the central part and ray florets in the peripheral part (arrows); (**B**) ray florets with visible serration in the apical part (arrows); (**C**) fragment of the epidermis surface in the central part of the ray floret with visible cuticular crests on the cell walls (arrowheads); (**D**) epidermis surface in the apical part of the ray floret; (**E**,**F**) fragments of the epidermis surface in the lower part of the ray floret with visible longitudinal cuticular striae and secretion residues (asterisks). Scale bars: 0.5 cm (**A**,**B**), 20 μm (**D**,**E**), 10 μm (**C**,**F**).

The investigated *C. cyanus* inflorescences (Figure 1A) were composed of 8–11 ray florets and 15–23 disc florets. The length of the funnel-shaped ray florets consisting of fused petals (Figure 1B) was 14–18 mm, and their widest diameter was in the range of 9–14 mm. The apical part of the ray florets had 7–9 lobes (Figure 1A).

The adaxial and abaxial epidermis of the ray florets was composed of elongated cells, with the central part of the surface exhibiting characteristic folding of the crested cuticle visible in the scanning electron microscope (Figure 1C). The epidermis cells in the lobes of the ray florets were shorter, and the cuticle folds on their surface formed a crested pattern (Figure 1D). In turn, the surface of the epidermis cell walls in the lower part of the ray florets was characterised by the presence of massive cuticle bands but no crests (Figure 1E,F). 

The scanning electron microscopy (SEM) revealed secretion deposits in some areas of the petal epidermis surface (Figure 1 E,F). No trichomes or papillae were found in the ray floret epidermis.

### 2.2. Secretory Activity of the Epidermis and Parenchyma

**Figure 2:**
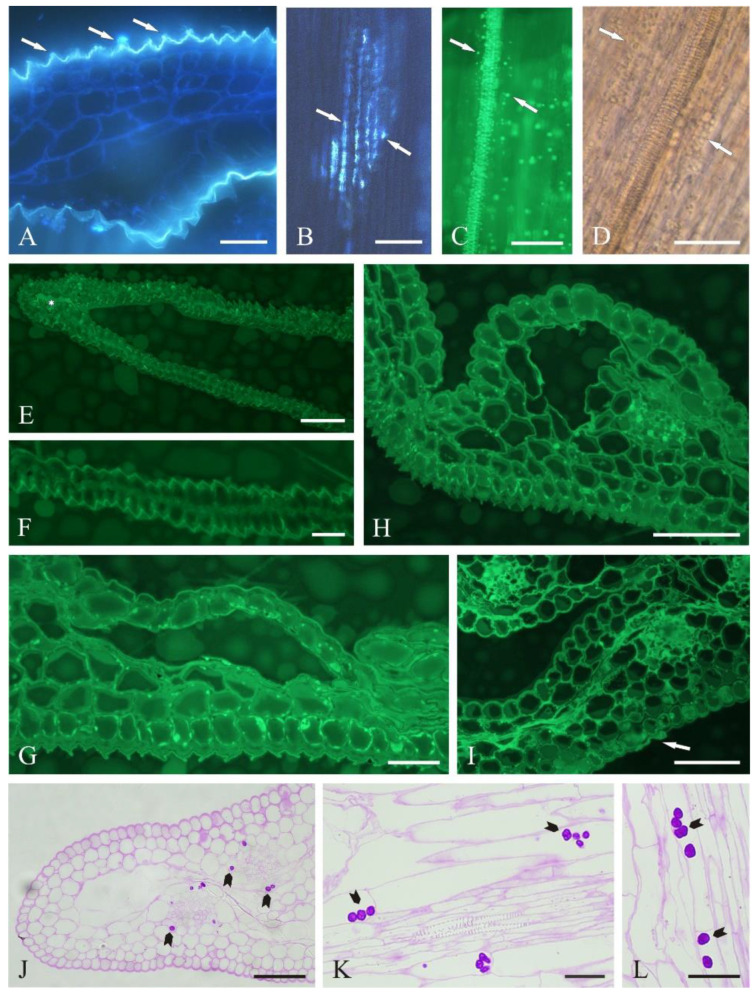
Fragments of *Centaurea cyanus* ray florets upon application of fluorescence and histochemical assays: (**A**) light blue fluorescence of the cuticle and secretion (arrows) on the cross section of the fresh ray floret; (**B**) autofluorescence of secretion (arrows) on the epidermis surface in the ray florets in top view; (**C**) secondary green fluorescence of tracheal elements and lipid droplets visible on the epidermis surface in the ray floret (arrows) after application of auramine; (**D**) orange-stained lipid droplets (arrows) in epidermis cells after treatment with Sudan III; (**E**–**I**) secondary green fluorescence of lipids visible in cross sections of cells of various tissues of the ray floret after application of auramine; (**E**,**F**) fragments of the apical part of the floret; (**E**) secretion (star) is visible in the intercellular space; (**G**,**H**) fragments of the central part of the floret; (**I**) fragment of the lower part of the floret; remnants of secretion visible on the abaxial epidermis surface (arrow); (**J**–**L**) fragments of the cross sections of the ray floret with visible starch grains (arrowheads) in the cells of perivascular parenchyma after the PAS reaction; (**J**) cross section; (**K**,**L**) longitudinal section. Scale bars: 50 μm (**B**–**E**,**H**–**J**), 20 μm (**A**,**F**,**G**,**K**,**L**).

The cross sections of fused petals observed using the fluorescence microscope exhibited light blue autofluorescence of the epidermis cell cuticle. In some areas, secretion-containing vesicles in the distended cuticle emitted light blue fluorescence (Figure 2A). The blue, fluorescent secretion was also visible on the surface of the epidermis cells of fresh petals, where it formed linear clusters along the cuticle crests (Figure 2B).

Lipid droplets emitting light green secondary fluorescence after the auramine treatment were visible on the surface of the petals, especially near the vascular bundles (Figure 2C). The Sudan III treatment confirmed the lipid nature of the orange-stained droplets secreted by the epidermis cells (Figure 2D). Fluorescence was also emitted by the secretion present in the intercellular spaces in the parenchyma and visible on the cross sections in permanent slides viewed under the fluorescence microscope after the auramine treatment (Figure 2E–H). Fluorescence of secretion residues on the surface of epidermis cells was observed as well (Figure 2I).

Starch grains are often observed in the cells of secretory tissues. We demonstrated their presence in parenchyma cells located around vascular bundles after the PAS reaction (Figure 2J–L).

### 2.3. Ultrastructure of Secretory Cells

**Figure 3:**
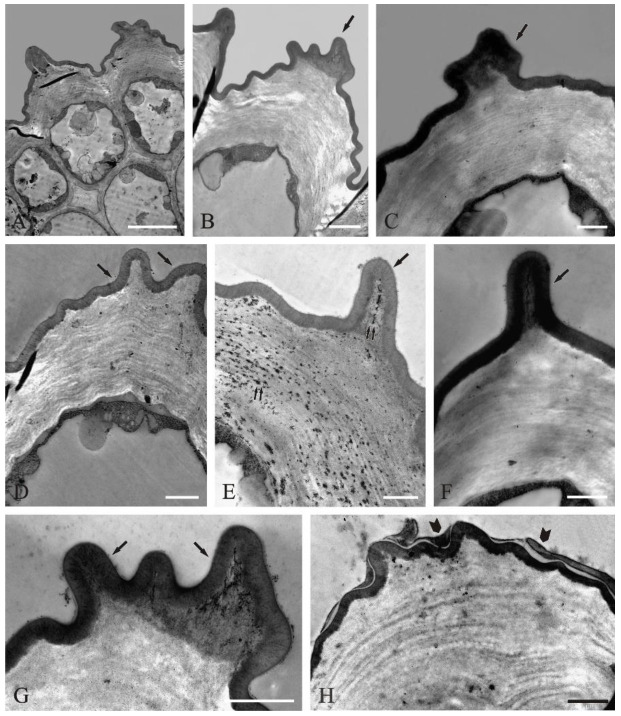
Ultrastructure of epidermis cells in the central part of *C. cyanus* ray florets (transmission electron microscopy-TEM): (**A**) epidermis cells and subepidermal parenchyma cell fragments in cross section; the epidermis cells have a very thick outer cell wall, large vacuoles, and parietally located cytoplasm; (**B**–**F**) fragments of epidermis cells with a very thick outer wall and cuticular crests in its apical part (arrows); (**D**,**E**,**H**) layered arrangement of structural elements in the cell wall; (**E**) an osmophilic, flocculent substance, possibly the released secretion, visible in the wall (double arrows); (**B**,**C**,**F**,**G**) dark substance/secretion accumulated in the wall under the cuticle in the crest area; (**H**) a dark layer of secretion residues visible on the cuticle surface (arrowheads). Scale bars: 5 μm (**A**), 2 μm (**B**), 1 μm (**C**–**H**).

Both the epidermis and the parenchyma in the *C. cyanus* ray flowers served the secretory function. The outer wall of the epidermis cells had a considerable thickness (3.1 µm) and was covered by a relatively thin layer of a folded cuticle (0.36 µm) forming crests in the apical part (Figure 3A–C). The central part of the cells was occupied by a large vacuole, and the dense cytoplasm was located parietally (Figure 3A–D). The cytoplasm contained numerous ribosomes, mitochondria, smooth endoplasmic reticulum profiles, plastids, and numerous vesicles (Figure 3B,D). The outer wall had a multi-layer structure (Figure 3D–F,H), while the cuticle was formed by a homogeneous layer with microchannels (Figure 3D–G). In some areas of the cell wall, there were dark, flocculent structures, which were likely part of the released secretion (Figure 3E). This osmophilic substance accumulated mainly in the subcuticular part of the crests (Figure 3E–G), and was then likely released through the cuticle microchannels and formed a layer with varying thickness on the surface (Figure 3H).

**Figure 4:**
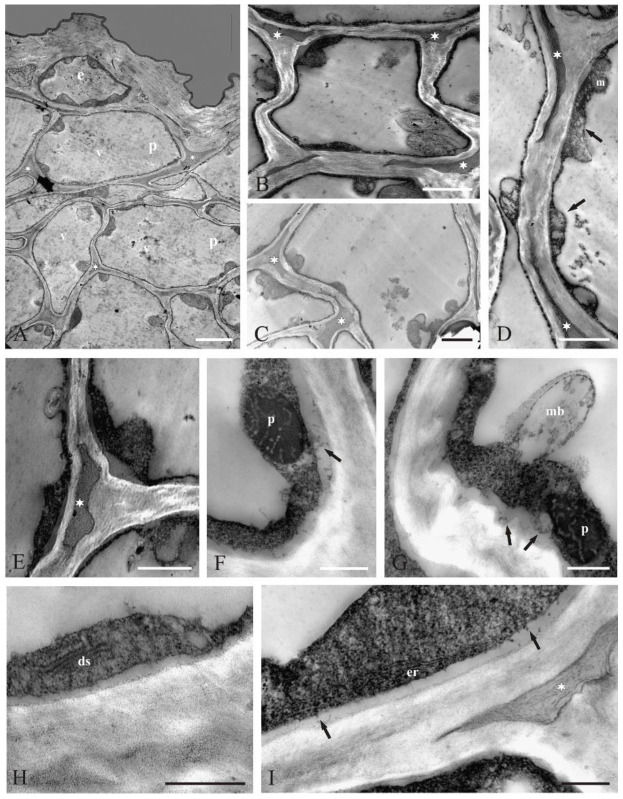
Ultrastructure of parenchyma cells in *C. cyanus* ray florets (TEM): (**A**) under the epidermis (e)-parenchyma cells (p) with different-shaped large vacuoles (v), parietal cytoplasm and large intercellular spaces (asterisks) with a dark substance; (**B**) a cell with walls with varying thickness containing multivesicular bodies in the vacuole and parietally located cytoplasm. The intercellular spaces (asterisks) are filled with a dark substance/secretion; (**C**) large intercellular spaces filled with a dark substance/secretion along the entire length of some walls (asterisks); (**D**) fragments of cells with numerous different-sized vesicles (arrows) and mitochondria (m) in the cytoplasm and secretion in the intercellular spaces (asterisks); (**E**) fragments of cells with dense cytoplasm and a heterogeneous substance/secretion in the intercellular space (asterisk); (**F**) a plastid (p) with dark stroma, tubular internal structure, and small vesicles in the periplasmic space (arrow); (**G**) multivesicular body (mb) directed towards the vacuole, plastid (p) with dark stroma and small vesicles in the periplasmic space (arrows); (**H**) dictyosome (ds) in the dense cytoplasm; (**I**) smooth endoplasmic reticulum (er) profile and numerous small vesicles located close to the plasmalemma (arrows) and fibrillar material in the intercellular space (asterisk). Scale bars: 5 μm (**A**), 2 μm (**B**–**D**), 1 μm (**E**), 0.5 μm (**F**–**I**).

The secretory parenchyma cells had irregular shapes (Figure 4A,B). Large intercellular spaces filled with a dark heterogeneous substance were visible between the cells (Figure 4B–E). As in the case of the epidermis cells, the cytoplasm formed a thin parietal layer, and the central part of the cells was occupied by large vacuoles (Figure 4A–C). The dense cytoplasm contained numerous mitochondria (Figure 4D), ribosomes (Figure 4F–I), and plastids with dark stroma and well-developed internal tubules (Figure 4F,G). Additionally, there were dictyosomes, numerous different-sized vesicles (Figure 4H), and endoplasmic reticulum profiles mainly located close to the plasmalemma (Figure 4I). Multivesicular bodies, likely originating from the cytoplasm, were visible in the vacuoles (Figure 4B,G).

### 2.4. Volatile Compounds

**Figure 5:**
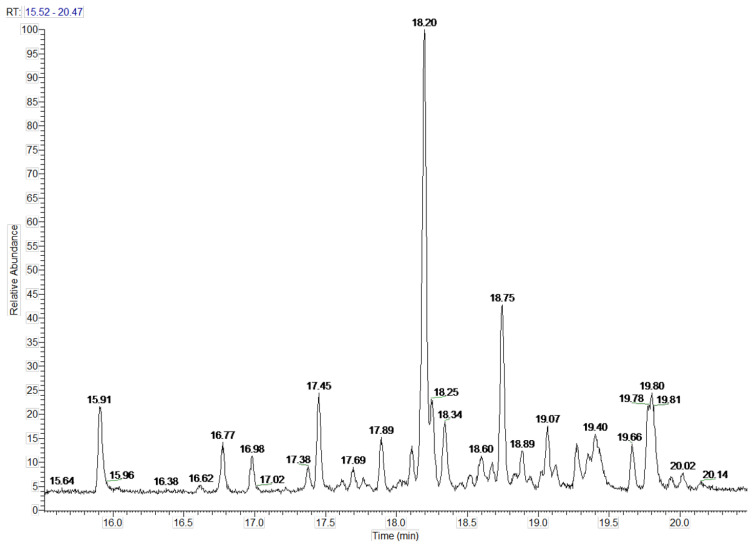
Separation of *Centaurea cyanus* L. fresh flowers volatile compounds.

In the petals of *C. cyanus* ray florets, 31 volatile components were detected, and 25 (94.74%) of the compounds were identified (Figure 5, Table 1 and Table 2). The other six components (5.26%) were partially characterised based on their retention indices and retention times. All volatile compounds represented terpenes, with one oxygenated monoterpene (5.22%) and 24 sesquiterpenoids (89.52%) including 15 bicyclic sesquiterpenes. Beta-caryophyllene (26.17%), alpha-humulene (9.77%), cis-calamenene (5.25%), alpha-copaene (4.98%), epi-zonarene (4.45%), beta-cedrene (4.21%), and delta-cadinene (4.05%) were the predominant constituents of the sesquiterpenoids. Only one monoterpene, i.e., bornyl acetate (5.22%), was detected.

## 3. Discussion

### 3.1. Secretory Activity of Corolla Tissues

No such secretory structures as papillae, glandular trichomes, and ducts were detected in the *C. cyanus* ray florets. The absence of secretory ducts in floret petals was reported in several other species from the family Asteraceae, e.g., *Matricaria chamomilla* [16], *Santolina ligustica* [15], *Centaurea rupestris*, and *C. fritschii* [14]. However, these *Centaurea* species had large biseriate glandular hairs on the corolla surface. A similar type of trichomes was detected on the corolla in other species of this family: *Helichrysum stoechas* [32], *Chamomilla recutita* [16,33], and *Inula helenium* [34]. Centrally located disc florets in *C. cyanus* inflorescences were found to have only papillae [20]. Papillae on disc florets were also observed in other genera of this family: *Flourensia* [35] and *Petasites* [36]. 

The present study demonstrated that the epidermis cells of petals in the *C. cyanus* ray florets released fragrant secretory products. Floral tissues secreting such products, i.e., osmophores, produce trichomes and papillae in the epidermis, or the epidermis is composed of cells with a simple cubic form [37]. The epidermis of *C. cyanus* ray florets is an osmophore similar to the latter type. 

The outer wall of the epidermis cells in the *C. cyanus* ray florets (tribe *Cardueae*) exhibited a crested cuticular pattern, which was also observed in an earlier study [20]. The present comprehensive study demonstrated the presence of the pattern only in the apical and central parts of these florets. In turn, longitudinal cuticular striation of the epidermis surface was observed in the lower part of the ray florets. The crested cuticular pattern has also been observed in Asteraceae species belonging to other tribes, e.g., *Cichorieae* and *Mutisieae* [38,39].

As shown in the cross sections, the outer cell walls of the petal epidermis cells were very thick (3.1 µm), and there were microchannels in the cuticle layer. The secretion was released through the wall in the area of the cuticular crests, as shown by fluorescence microscopy and transmission electron microscopy (TEM). The secretion transported via vesicles from the distal part of the cell to the periplasmic space reached the epidermis surface by crossing the cell wall and microchannels in the cuticle. A similar transport of aromatic secretions via microchannels into the environment was observed in the osmophores of *Orbea variegata* [40] and *Passiflora superosa* [41,42]. However, cuticular diffusion related to the lipophilic nature of the cutin is recognized as the most common way of releasing fragrance by plants [37,43,44]. Some plants (*Acianthera*) have been described to release secretory products (e.g., essential oil) through stomata [45].

In the epidermis cells of the fresh ray florets, different-sized lipid drops were shown by both the light microscope (Sudan III) and the fluorescence microscope. In turn, on the surface of the epidermis of these florets, residues of a copious secretion covering the outer cell wall were shown in the fixed material by SEM and TEM. The presence of the secretion was likely associated with its high viscosity and the fact that not all of its components were volatilised. Therefore, it can be assumed that *C. cyanus* ray florets secrete resinaceous material.

The cells of the subepidermal parenchyma of the ray florets had many features of scent-producing cells: high cytoplasmic density, cytoplasm rich in ribosomes, smooth endoplasmic reticulum, plastids with dark stroma, numerous lipid droplets, dictyosomes, many mitochondria, and vesicles, which were often localised near the plasmalemma. These traits are recognised as characteristic for osmophores by many authors [41,44,46]. The smooth endoplasmic reticulum profiles in the *C. cyanus* flower cells were located near the plasmalemma in the peripheral part of the cytoplasm. As reported by Skubatz et al. [47], endoplasmic reticulum (ER) structures in *Sauromatum* can associate with the cell membrane during secretion of volatile sesquiterpenes. The presence of large intercellular spaces in the parenchyma containing heterogeneous lipid material, likely the secretion, should also be regarded as one of the traits of glandular tissues.

The presence of a lipid-containing substance in the intercellular spaces of the parenchyma in the ray florets indicates the apoplast route of secretion in these flowers. As reported by other authors, lipid droplets may be transported in small vesicles to periplasmic and intercellular spaces [45]. In many plants, the exchange of substances between secretory cells occurs via vesicles and/or via plasmodesmata (symplast route) [37]. In the case of the *C. cyanus* ray florets, there were no plasmodesmata in the parenchyma cell walls.

It was shown previously that lipids, which are components of such a secretory product as essential oil, are formed in plastids [37,48]. Other authors have reported that secretion/essential oil droplets often originate from plastids, periplastidal reticulum, and smooth reticulum but sometimes from dictyosomes or other organelles [11]. The connection between the metabolic pathways in the cells of various organs and the site of terpenes synthesis indicates that plastids are mainly responsible for the formation of volatile mono-(C_10_) and diterpenes (C_20_) [49]. In turn, the cytosol, ER, and peroxisome pathway leads to the formation of precursors of volatile sesquiterpenes (C_15_) [3,50,51].

### 3.2. Volatile Compounds of Petals

Most literature data present the composition of essential oil in different *Centaurea* species obtained from aerial plant parts [26,28,29,30]. Our research is the first to show the composition of the most volatile compounds collected from *Centaurea cyanus* fresh corolla of flowers. 

As shown by the literature data, sesquiterpenoids are the main class of essential oil components in various *Centaurea* species [26,28,29,30]. Our phytochemical study has demonstrated that, among volatile compounds present in fresh petals of *Centaurea cyanus*, there are 89.52% of sesquiterpenoids (24 sesquiterpenoids) and 5.22% of oxygenated monoterpenes. Considering the first group of solutes, the highest content was determined for β-caryophyllene (26.17%), followed by α-humulene (9.77%), *cis*-calamanene (5.25%), α-copaene (4.98%), *epi*-zonarene (4.45%), β-cedrene (4.21%), δ-cadinene (4.05%), and β-copaene (3.74%). The content of the other terpenoids was in the range from 2.83% (γ-muurolene) to 0.45% (α-calacorene). Oxygenated monoterpenes were represented only by bornyl acetate (5.22%) in our sample.

β-caryophyllene has been reported by various authors as an important essential oil component from *Centaurea* species growing in Asia. The content of this compound is species-dependent and varies from 8.1% (in *C. pseudoscabiosa* subsp. *pseudoscabiosa*) to 33.9% (*C. depressa*) [31,52,53,54,55]. A high concentration of β-caryophyllene in essential oil obtained from *Centaurea ragusina* flowers was also presented by Politeo [26]. 

α-humulene, α-copaene, and δ-cadinene have also been detected in *Centaurea ragusina* flowers [26] and extracts of *Centaurea intricate* flowering aerial parts [28]. Furthermore, some authors suggest that cadinene and its isomers are the most popular sesquiterpenes in plant essential oils [56]. 

It is remarkable that only one monoterpene derivative (bornyl acetate) was detected in the investigated volatile compounds. This compound does not occur in *Centaurea* species growing in the Middle East (Egypt or Iran) [26,28,29,31,52,53,54,55]. However, it can be found in plants originating from Tatarstan (*Centaurea scabiosa*. flowers) [57] or the central Balkans (*Centaurea orientalis* and *Centaurea atropopurpurea*) [58]. Thus, the presence of bornyl acetate in *Centaurea* plants seems to be dependent on the latitude. This compound has also been detected in the resin of two species of the *Taxodium* genus [59].

Many of the other volatile compounds (α-copaene, β-elemene, β-caryophyllene, β-copaene, cis-muurola-3,3-diene, α-humulene, γ-cadinene, δ-cadinene, α-cadinene) from the Polish *C. cyanus* detected by GC-MS have been reported to occur in *C. orientalis*, *C. atropurpurea* f. *flava*, and *C. atropurpurea* plants growing in the central Balkans [58]. 

Detailed data on the biological activity of *Centaurea cyanus* and other flower extracts from *Centaurea* species have been presented lately by Sharonova et al. [57]. Antimicrobial and antioxidant effects have been shown in the case of *Centaurea cyanus* L. from Kosovo (antimicrobial activity) [60] and Romania (antioxidant properties) [61]. 

In the present study, we have shown that sesquiterpenes are the main group of components in the volatile compounds from *C. cyanus* flowers. Literature data show that, together with sesquiterpene lactones and monoterpenes, sesquiterpenes are characteristic constituents of essential oil and resin in the family Asteraceae. Hence, these compounds may be important for the chemotaxonomy of this family [62,63]. The results of the present study are, to a certain extent, similar to the data on other taxa from this family.

In our studies, the secretion in *C. cyanus* flowers forms large clusters on the petal epidermis surface visible in scanning electron microscopy. These remnants of the secretory product may be sesquiterpenes, which are semi-volatile compounds that are characterised by low volatility and easily adhere to plants’ surfaces, as reported in the literature [1]. 

As reported by Parimal et al. [64], caryophyllene and δ-cadinene are sesquiterpenes that occur frequently in plant resins. These two compounds were found to contribute significantly (β-caryophyllene 26.17%, δ-cadinene 4.05%) to the composition of the volatile compounds in the *C. cyanus* flowers. In the *C. cyanus* flowers, we found 14 sesquiterpenes (β-caryophyllene, α- cubebene, α-ylangene, α- copaene, β-elemene, β-copaene, aro-madendrene, α-humulene, trans-cadina-1(6)-4-diene, γ-muurolene, cis-cadina-1,4-diene, γ-cadinene, δ-cadinene, α-cadinene) identical with those identified in oleoresin isolated from *Copaifera multijuga* by Silva et al. [65]. This fact may indicate that the secretion determined in *C. cyanus* flowers is oleoresin as well.

Additionally, it is known that the odoriferous secretion released by organs of various plant species frequently contains essential oil associated with resins or gums [9,64]. This seems to be the case of *C. cyanus* flowers, as their secretion exhibits the characteristics of a resinaceous substance.

## 4. Materials and Methods

### 4.1. Plant Material

Ray florets from *Centaurea cyanus* inflorescences were used as the material for the microscopic and phytochemical studies. These flowers are sterile and only consist of fused petals. The plant material was collected by Aneta Sulborska-Różycka at the beginning of flowering in July 2019 from fields in Motycz (administrative district of Gmina Konopnica, within Lublin County, Lublin Voivodeship, in eastern Poland; geographical coordinates N: 51.239170, E: 22.379440).

The botanical identification of the plants was made by taxonomy specialist Professor Bożena Denisow. Collected plant specimens were also compared with authentic samples (no 123) deposited in the Department of Botany and Plant Physiology, University of Life Sciences in Lublin (Poland).

Fresh ray florets (30) were randomly chosen for the morphometric analyses from 20 inflorescences.

### 4.2. Light Microscopy

The material for microscopic examinations consisted of 10 florets chosen randomly from 10 inflorescences. Handmade cross sections of the lower, middle, and upper parts of the fresh florets were prepared for preliminary analyses using razor blades. Observations of semi-permanent slides prepared in glycerin with water (1:1) were carried out with the use of a Nikon Eclipse 400 light microscope. An alcoholic Sudan III solution (POCH, Gliwice, Poland) was used for detection of total lipids in the fresh material [66].

Semi-thin sections were prepared from the same areas of the ray florets. Fragments of corollas (5 × 5 mm) were fixed in 2.5% glutaraldehyde in phosphate buffer (pH 7.2; 0.1 M) for 12 h at 4 °C. In the next step, the samples were washed three times in phosphate buffer, dehydrated in an ethanol series, and embedded in LR white resin (LR white acrylic resin, medium grade, Sigma-Aldrich, Saint-Louis, MO, USA). The semi-thin sections (0.7–0.9 μm) were cut longitudinally and transversally using a Reichert Ultracut S ultramicrotome (C. Reichert Optische Werke AG, Vienne, Austria) and a glass knife. For general histology, the sections were stained with a 1% (*w*/*v*) aqueous methylene blue-azure II solution [67]. The presence of insoluble polysaccharides was detected using Periodic Acid-Schiff’s (PAS) staining [67] after blocking free aldehyde groups. The sections were examined using an Olympus CX 23 light microscope (Olympus, Tokyo, Japan) equipped with an Olympus EP50 digital camera (Olympus) and EPview software.

### 4.3. Fluorescence Microscopy

The autofluorescence of ray floret tissues and sections was observed using a Nikon Eclipse 90i fluorescence microscope (Nikon, Tokyo, Japan) with UV-2 B filter equipped with a digital camera (Nikon Fi1) and NIS-Elements Br 2 software. Secondary lipid fluorescence was investigated after treatment of the semi-thin sections with auramine O [68]. The staining reaction was examined using a fluorescein isothiocyanate filter. Control sections were used in each case.

### 4.4. Transmission Electron Microscopy (TEM)

Sections (4 × 4 mm) from different ray florets (n = 5) were fixed as described above and treated for 1.5 h with a 1% osmium tetraoxide solution at 0 °C. The samples were washed with distilled water, and graded ethanol series were used for dehydration. Next, the plant material was saturated in 1:3, 1:1, and 3:1 mixtures of LR White resin (LR white acrylic resin, medium grade, Sigma-Aldrich, Saint-Louis, MO, USA) and acetone for 3 h each. After embedding in LR White resin, the samples were cut into ultra-thin sections (from 60 to 90 mm) with the use of the Reichert Ultracut Microtome. The ultrastructure of the cells was observed with the use of a JEM 1400 (JEOLL Ltd., Tokyo, Japan) transmission electron microscope at an accelerating voltage of 120 kV equipped with an 11 Megapixel TEM Camera MORADA G2 (EMSIS GmbH, Münster, Germany). 

### 4.5. Scanning Electron Microscopy

For observations of the epidermis surface, small pieces of ray florets (about 3 × 3 mm) were fixed in a 2.5% or 4% glutaraldehyde solution in 0.1 M phosphate buffer (pH 7.0) at room temperature for 2 h and then washed in phosphate buffer four times at 20-min intervals. The fixed plant material was dehydrated in graded ethanol series and immersed in absolute ethanol (POCH, Gliwice, Poland) three times for 30 min. Next, the floret samples were critical-point dried in liquid CO_2_ using a K 850 Critical Point Dryer and sputter-coated with gold (20 mm thickness) using K 550X (Emitech, Ashford, UK). The observations were carried out using a Tescan Vega II LMU scanning electron microscope (Tescan, Brno, Czech Republic) at an accelerating voltage of 30 kV [69].

### 4.6. Determination of Volatile Compounds in the Flowers 

#### 4.6.1. SPME Extraction of Floral Volatile Components

A sample (2 g) of freshly collected flowers of *C. cyanus* was introduced into the SPME (Solid Phase Microextraction) glass thimble. The SPME sorption fibre (made from polydimethylsiloxane, PDMS, diameter 30 μm, Sigma-Aldrich, Saint-Louis, MO, USA) was slotted into the shredded raw material for 30 min. The extraction was performed at room temperature. Next, the system was placed in the injector of a gas chromatograph (description presented in part GC-MS determination), where the thermodesorption (at 250 °C for 3 min) took place. The volatile compounds were then separated on a chromatographic column. A dispenser was used to perform the determinations (split 1:50; T = 250 °C). The temperature program for SPME was as follows: 35 °C for 3 min and then 8 °C/min to 250 °C (final isotherm 1 min).

#### 4.6.2. GC-MS determination

The composition of the *Centaurea cyanus* L. volatile compounds was determined with the GC-MS technique (ITS-40 apparatus (GC/ITMS system), Finnigan MAT, Temecula, CA, USA) with an RT-5 capillary column (Resteck, Bellefonte, PA, USA) 20 m long, 0.18 mm in diameter, and stationary phase film thickness of 0.25 μm. The MS analysis was performed using electron ionization (70 V) with a 35–500 *m*/*z*.

Qualitative analysis was carried out by comparison of the MS spectra with the NIST spectral library (National Institute of Standards and Technology, Gaithersburg, MD, USA) [70] and the spectral library of the UMCS Analytical Laboratory (Lublin, Poland). Identity of the volatile compounds was confirmed by their retention indices, which were determined in relation to a homologous series of n-alkanes (C_7_–C_18_). The quantitative composition of volatile compounds was determined assuming that the sum of individual compounds is 100%.

## 5. Conclusions

This study is the first report on the constituents of volatile compounds in fresh petals of *C. cyanus* flowers (ray florets). The main group of aroma components was constituted by sesquiterpenes, with the highest content of β-caryophyllene and α-humulene.

The microscopic analyses facilitated localisation of the sites of secretion production, i.e., parenchyma cells. The secretion was transferred via vesicles to the cells of this tissue and intercellular spaces and then to epidermal cells. Next, it penetrated the surface of these cells through cuticle microchannels, most often in the crested pattern zone. The present study showed the function of the crested cuticle present in the flowers of some Asteraceae, which was involved in the release of secretory products in *C. cyanus*.

The physical properties and chemical composition of the secretion suggest that cornflower flowers secrete a mixture containing a resinaceous substance.

## Figures and Tables

**Table 1:** Qualitative and quantitative composition of volatile compounds in petals of cornflower (*Centaurea cyanus* L.).

No	Compound	Retention Index *	Retention Time [min]	Percent [%]
Oxygenated monoterpenes				
1	Bornyl acetate	1290	15.91	5.22
		Total percent		5.22
Sesquiterpenoids				
1	bicycloelemene	1342	16.77	2.56
2	α-cubebene	1354	16.98	1.98
3	α-ylangene	1378	17.38	1.21
4	α-copaene	1383	17.45	4.98
5	β-elemene	1397	17.69	1.26
6	cyperene	1410	17.89	2.74
7	α-cedrene	1424	18.11	2.01
8	β-caryophyllene	1429	18.20	26.17
9	β-cedrene	1432	18.25	4.21
10	β-copaene	1438	18.34	3.74
11	aromadendrene	1450	18.52	0.71
12	α-humulene	1464	18.75	9.77
13	cis-muurola-3,5-diene	1455	18.60	1.79
14	trans-cadina-1(6)-4-diene	1482	19.02	0.70
15	γ-muurolene	1485	19.07	2.83
16	α-muurolene	1489	19.13	1.18
17	α-selinene	1497	19.27	2.51
18	cadina-1,4-diene	1503	19.35	1.98
19	epi zonarene	1506	19.40	4.45
20	γ-cadinene	1523	19.66	2.20
21	δ-cadinene	1532	19.79	4.05
22	cis calamanane	1533	19.80	5.25
23	α-cadinene	1548	20.02	0.79
24	α-calacorene	1556	20.14	0.45
		Total percent		89.52
Unknown				
1		1332	16.61	0.55
2		1392	17.61	0.71
3		1402	17.77	0.62
4		1460	18.68	0.99
5		1473	18.89	1.88
6		1542	19.94	0.51
		Total percent		5.26

* The retention indices were determined in relation to a homologous series of n-alkanes (C_7_–C_18_).

**Table 2:** Chemical structures of the most important volatile compounds present in *Centaurea cyanus* flowers.

**Oxygenated Monoterpenes**						
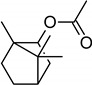 bornyl acetate						
**Sesquiterpenoids**						
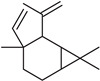	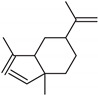	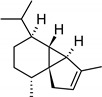	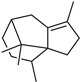	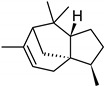	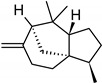	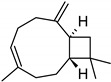
Bicycloelemene	β-elemene	α-cubebene	cyperene	α-cedrene	β-cedrene	β-caryophyllene
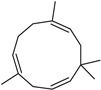	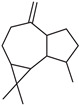	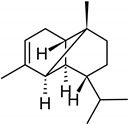	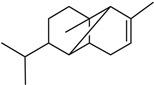	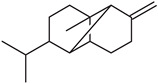		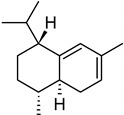
α-humulene	aromadendrene	α-ylangene	α-copaene	β-copaene		cis-muurola-3,5-diene
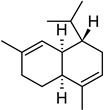	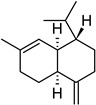	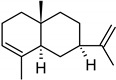	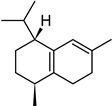	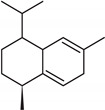	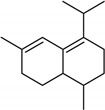	
α-muurolene	γ-muurolene	α-selinene	trans-cadina-1(6)-4-diene	cadina-1,4-diene	epi zonarene	
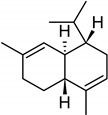	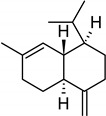		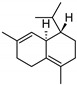	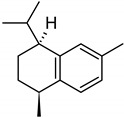	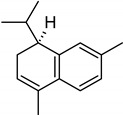	
α-cadinene	γ-cadinene		δ-cadinene	cis calamenene	α-calacorene	

## Data Availability

The data presented in this study are available on request from the corresponding author.

## Sample Availability

Samples are not available from the authors.
